# Comparative efficacy and safety of combination therapies for advanced melanoma: a network meta-analysis

**DOI:** 10.1186/s12885-018-5259-8

**Published:** 2019-01-09

**Authors:** Qing An, Zhihao Liu

**Affiliations:** 1 Jiangsu Cancer Hospital & Jiangsu Institute of Cancer Research & The Affiliated Cancer Hospital of Nanjing Medical University, Nanjing, China; 2 Institute for Health Education, Jiangsu Provincial Center for Disease Control and Prevention, Nanjing, China

**Keywords:** Advanced melanoma, PD-1/L1 blockade, CTLA-4 blockade, BRAF inhibitor, MEK inhibitor

## Abstract

**Background:**

Currently, the major treatment modalities of advanced melanoma are immune check point and mitogen-activated protein kinase (MAPK) pathway inhibitors. As lacking head-to-head randomizedcontrolled trials (RCTs) comparing immune check point and MAPK pathway inhibitors, we evaluated the efficacy and toxicity with different treatment combinations of immune check point or MAPK pathway inhibitors for advanced melanoma by network meta-analysis.

**Methods:**

We searched for RCTs in Pubmed, Embase, Ovid MEDLINE, Web of Science and Cochrane Central Register for Controlled Trials through March 2017. Two reviewers performed a network meta-analysis by assessing the hazard ratios (HRs) for overall survival (OS) and progression-free survival (PFS), as well as by evaluating serious adverse events (SAEs).

**Results:**

Twenty-four eligible RCTs involving 10,951 patients assigned to 11 treatment modalities were included. The combination of BRAF and MEK inhibitors demonstrated an improved OS benefit compared with all the other treatments except programmed death-1/ligand-1 (PD-1/L1) blockade because the difference in OS between the BRAF-MEK inhibitor combination and PD-1 blockade (HR: 0.85; 95% credible interval (CrI): 0.59, 1.21) was not significant. For PFS, the BRAF and MEK inhibitor combination showed a significant advantage compared with other treatments apart from the combination of PD-1/L1 and cytotoxic T lymphocyte-associated antigen-4(CTLA-4) blockade (HR:0.61; 95% CrI: 0.30, 1.25). The MEK inhibitor combined with chemotherapy was associated with the highest risk of SAEs (HR: 1.76 95% CrI: 1.21, 2.48).

**Conclusions:**

The combination of BRAF and MEK inhibitors exhibited a survival advantage in OS and PFS and comparable risk of toxicity compared with chemotherapy.

**Electronic supplementary material:**

The online version of this article (10.1186/s12885-018-5259-8) contains supplementary material, which is available to authorized users.

## Background

Melanoma is an aggressive form of cancer, with a high mortality rate and low 5-year survival rate in patients with advanced-stage disease [1]. However, since the development of immune check point inhibitors and targeted therapies, melanoma treatment has remarkably improved patient survival [2]. Immune checkpoint inhibitors, a group of monoclonal antibodies, mainly block co-stimulators that down-regulate T-cell function to help tumour cells escape from immune attacks, such as by the T lymphocyte-associated antigen-4 (CTLA4) and programmed death-1/ligand-1 (PD1/L1) signalling molecules, to activate anti-tumour immune responses [3–5]. Mitogen-activated protein kinase (MAPK) pathway blockades are another class of molecules that can be used to effectively treat advanced melanoma; mainly composing of BRAF and MEK inhibitors. BRAF inhibitors specifically target *BRAF* V600 mutations [6, 7], and MEK inhibitors block the downstream signal protein kinases of the MAPK pathway [8].

Recently, with the advancement of targeted therapy, more therapies have been combined, such as CTLA-4 or PD-1/L1 blockade plus chemotherapy, CTLA-4 blockade plus PD-1/L1 blockade, BRAF inhibitor plus MEK inhibitor, MEK inhibitor plus chemotherapy and other combination regimens, have been proven to show improvement in comparison with single-agent regimens [9–11]. For example, the ipilimumab plus dacarbazine group showed a higher overall survival (OS) rate for 3 years than the dacarbazine group (20.8% vs. 12.2%, respectively). The nivolumab plus ipilimumab group showed better median progression-free survival (PFS) than the ipilimumab group (11.5 months vs. 2.9 months, respectively) [10, 11]. Meanwhile, BRAF and MEK inhibitors also significantly improved the effectiveness of treatment and reduced the incidence of secondary skin cancer [12].

However, the evidence from several trials does not offer a holistic view for these two categories of treatments, because head to head randomized controlled trials (RCTs) are still lacking among different implements (PD-1/L1 blockade plus chemotherapy, CTLA-4 blockade plus chemotherapy, PD-1/L1 blockade plus CTLA-4 blockade, PD-1/L1 blockade plus adjuvant therapy, BRAF inhibitor plus MEK inhibitor and MEK inhibitor plus chemotherapy). Network meta-analysis (NMA) can integrate direct and indirect evidence from RCTs and perform indirect comparisons through a common comparator [13–16]. We used this tool to analyse the efficacy and toxicity of different combination regimens of immune check point inhibitors or MAPK pathway inhibitors by OS, PFS and serious adverse events (SAEs) in patients with advanced-stage melanoma.

## Methods

### Literature search strategy

Two investigators (Q.A. and Z.L.) searched Pubmed, Embase, Ovid MEDLINE, Web of Science and Cochrane Central Register for Controlled Trials until March 2017 with the restriction of language to English and using the following key words and Medical Subject Heading terms: advanced melanoma, immune check point inhibitor, CTLA-4 blockade, PD-1/ L1blockade, PD-1/L1blockade plus chemotherapy, CTLA-4 blockade plus chemotherapy, PD-1/L1 blockade plus CTLA-4 blockade, BRAF inhibitor, MEK inhibitor, BRAF inhibitor plus MEK inhibitor, BRAF inhibitor plus MEK inhibitor with PD-1/L1 blockade or CTLA-4 blockade; MEK inhibitor plus chemotherapy, ipilimumab, nivolumab, trametinib, cobimetinib, vemurafenib, dabrafenib and randomized clinical trials. We also reviewed the reference lists of published trials, relevant review articles, and conference (American Society of Clinical Oncology [ASCO], Annual Meetings and the European Cancer Conference [ECCO]) abstracts for other potential eligible trials. The electric search procedure followed the PRISMA (Preferred Reporting Items for Systematic Reviews and Meta-Analyses) guidelines and PRISMA Extension for Network Meta-analysis.

### Study eligibility

We included clinical trials according to the following criteria: (1) RCTs of adult patients with advanced melanoma (TNM stage III-IV); (2) treatments with combination regimens, such as PD-1/L1 blockade plus chemotherapy, CTLA-4 blockade plus chemotherapy, PD-1/L1 plus CTLA-4 blockade, CTLA4 blockade plus adjuvant therapy, PD-1/L1 blockade plus adjuvant therapy, BRAF plus MEK inhibitor, BRAF inhibitor plus MEK inhibitor with PD-1/L1 blockade or CTLA-4 blockade, MEK inhibitor plus chemotherapy, and BRAF inhibitor plus chemotherapy reporting at least one index of outcomes (OS, PFS and SAEs); and (3) published in English. Studies without a common comparator (such as a placebo or control arm) that provides connections through a network of different regimens were excluded. The most recent publication was applied to multiple publications of the same trial. Updated data were used when they were available. Two investigators (Q.A. and Z.L.) independently determined whether the trials met the inclusion criteria, with discrepancies resolved by consensus.

### Risk of bias

Q.A. and Z.L. evaluated the risk of bias for all the included studies using the Cochrane risk of bias tool [17]. Assessments were performed regarding sequence generation, allocation concealment, blinding, incomplete outcome data, and selective reporting. Three levels of risks (low, high and unclear) were reported for the included RCTs.

### Data extraction and outcome definitions

Two investigators (Q.A. and Z.L.) extracted the author name, journal name, year of publication, patient category, race, therapeutic regimens and clinical outcomes independently with predefined collection data sheets. The most interesting outcomes were OS and PFS in patients with advanced melanoma. The adverse and toxicity outcomes were abstracted from the main trial publications, supplemental appendices and relevant subsequent analyses.

### Statistical analysis

Since lacking head-to-head RCTs of comparing immune check point and MAPK pathway inhibitors, a Bayesian framework using the Marko chain Monte Carlo method was used to perform multiple treatment comparison network meta-analyses, including both direct and indirect RCT comparisons of the treatments. The network was constructed by comparing the major treatments: PD-1/L1 blockade plus chemotherapy, CTLA-4 blockade plus chemotherapy, PD-1/L1 blockade plus CTLA-4 blockade, CTLA-4 blockade plus adjuvant therapy, BRAF inhibitor plus MEK inhibitor and MEK inhibitor plus chemotherapy. The comparative effectiveness of the treatments regarding OS and PFS was summarized using the hazard ratio (HR) and corresponding 95% credibility intervals (CrIs). The inconsistency of the network meta-analysis was evaluated using the node-splitting technique, which evaluated the agreement between direct and indirect sources of evidence. Heterogeneity across studies was also evaluated. Ranking the different treatments in terms of their likelihood of showing the best results was performed using the P-score for each outcome [18]. Statistical analysis was performed using WinBugs (MRC Biostatistics Unit) and R software (Version 3.2.4) with the packages ‘netmeta’ and ‘pcnetmeta’ (version 0.8).

## Results

**Fig. 1:**
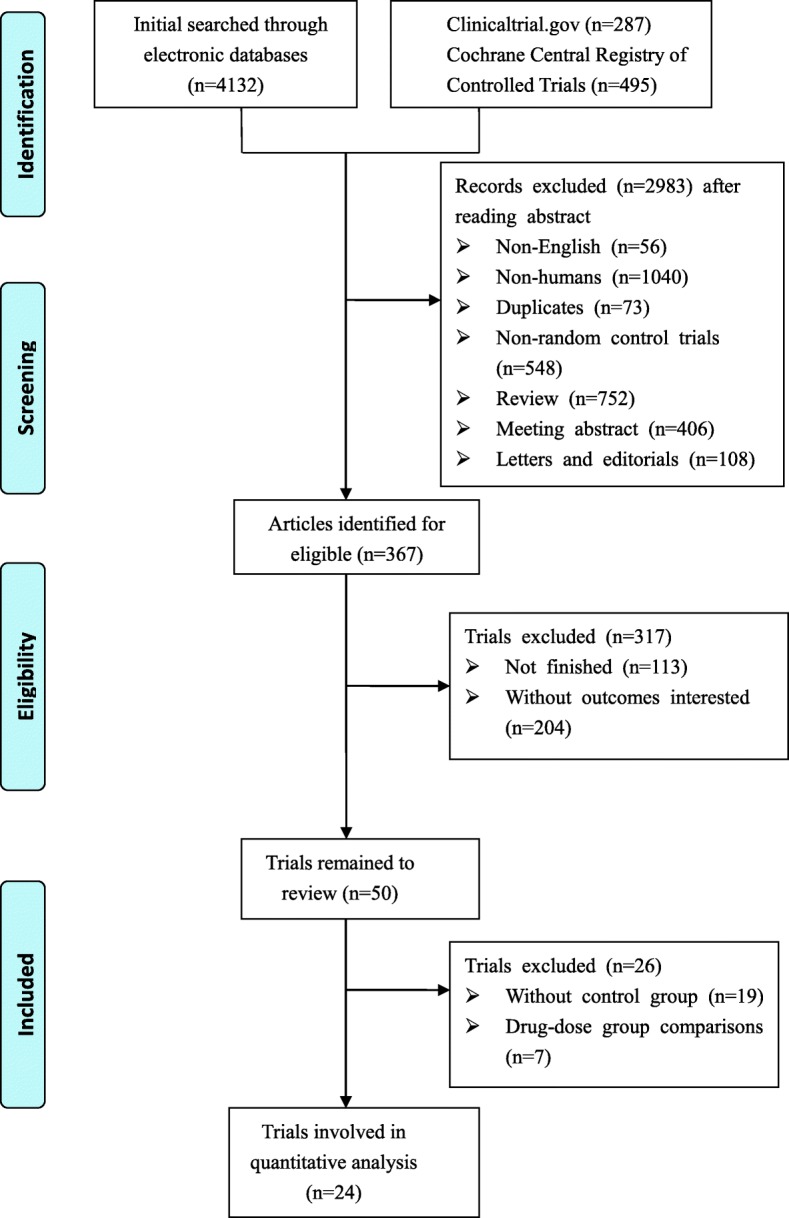
Diagram of the eligible study selection procedures

Three hundred sixty-seven relevant references were identified for review of their titles and abstracts. Of these, 50 randomized controlled trials were retrieved for more details (19 lacked a control group and were 7 drug-dose comparisons); finally, 24 phase II or III randomized controlled trials were identified that met the eligibility criteria of this study. In total, 10,951 patients were included in this network meta-analysis. Figure 1 shows the flow diagram of the literature search and selection of clinical trials. The characteristics of the 24 included trials are summarized in Table 1 [10–12, 19–39]. Four were three-arm trials, and the others were two-arm trials in this analysis. Additional file 1 shows more detailed information of trials as PD-L1 and *BRAF*V600 expressions and strategies that were used in this study, such as the combination of PD-1/L1 blockade and CTLA-4 blockade (*N* = 1087), combination of CTLA-4 blockade and chemotherapy (*N* = 502), combination of CTLA-4 blockade and adjuvant (*N* = 921), combination of BRAF and MEK inhibition (*N* = 1622), combination of BRAF inhibition and chemotherapy (*N* = 925), combined MEK inhibition and chemotherapy (*N* = 1377) and others.

**Table 1:** Baseline characteristics of included patients

First Author Year	No. of patients Treatment/Control	Age(median) Treatment/Control	Male sex (%)	Prior systemic therapy (%)		
			Treatment/Control	Type	Treatment	Control
PD-1/L1 versus chemotherapy						
Robert 2015	210/208	64/66	57.6/60.1	Adjuvant therapy	15.2	17.3
				Neoadjuvant therapy	0.5	0.5
Weber 2015	272/133	59/62	65/64	Ipilimumab	> 99	100
				Chemotherapy	53	54
				Vemurafenib	18	17
				Other immunotherapy	14	26
Ribas 2015	180/181/179	62/60/63	58/60/64	Ipilimumab	100/100	100
				Chemotherapy	50/46	48
				Other immunotherapy	37/34	31
CTLA-4 versus chemotherapy						
Ribas 2013	328/327	57/56	58/56	Radiation therapy	2	2
				Adjuvant therapy	2	3
CTLA-4 versus placebo						
Eggermont 2015	475/476	51/52	62/62	Surgical therapy	100	100
PD-1/L1 versus CTLA-4						
Schachter 2017	279/277/278	61/63//62	57.7/62.8/58.3	BRAF/MET inhibitor	18/16	20
				Immunotherapy	3/3	4.3
				Chemotherapy	13/15	10
CTLA-4 plus chemotherapy versus chemotherapy						
Robert C 2011	250/252	58/56	60.8/59.1	Adjuvant therapy	26	27
CTLA-4 plus adjuvant therapy versus CTLA-4 or adjuvant therapy						
Hodi FS 2010	403/137/136	56/57/56	61.3/59.1/53.7	Adjuvant therapy	22	23/24
				Systemic therapy	100	100/100
Hodi FS 2014	123/122	61/64	69.1/63.9	Adjuvant therapy	15	14
				Systemic therapy	31	30
CTLA-4 plus PD-1/L1 versus CTLA-4 or PD-1/L1						
Hodi 2016	95/47	64/67	66/68	Not mentioned		
Larkin 2015	314/315/316^1^	59/61/59	65.6/64.1/63.9	Not mentioned		
BRAF plus MEK versus BRAF						
Larkin 2014	247/248	56/55	59/56	Not mentioned		
Long 2014	211/212	55/56.5	53/54	Immunotherapy	27	29
Robert 2015	352/352	55/54	59/51	Immunotherapy	17	26
MEK plus chemotherapy versus chemotherapy						
McDermott 2008	51/50	55/60	75/66	Surgery	100	100
				Radation therapy	29	22
				Immunotherapy	67	72
Hauschild 2009	135/135	56/55.1	62/64	Surgery	100	100
				Radation therapy	27	33
				Chemotherapy	67	62
				Immunotherapy	26	30
Flaherty 2013	410/413	61/59	66/61	Immunotherapy	37	38
Robert 2013	45/46	57/52	49/61	Surgery	89	83
				Radation therapy	18	26
				Chemotherapy	7	2
				Immunotherapy	42	48
Gupta 2014	41/42	62/63	76/64	Not mentioned		
BRAF versus chemotherapy						
McArthur 2014	187/63	53/50	60/59	Radation therapy	20	16
				Immunotherapy	28	24
Hauschild 2012	337/338	56/52.5	59/54	Not mentioned		
MEK versus chemotherapy						
Flaherty 2012	214/208	55/54	56/49	Immunotherapy	32	28
				Chemotherapy	67	65
Kirkwood 2012	104/96	57.1/57	52.9/67.7	Not mentioned		
Carvajal 2014	50/51	62/62	52/62	Immunotherapy	16	22

### Overall survival

**Fig. 2:**
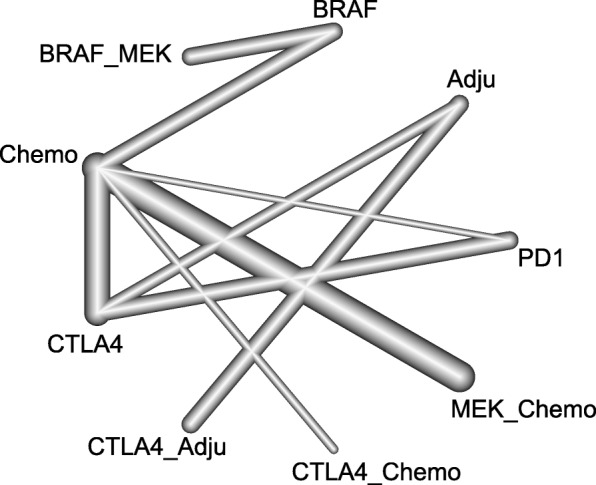
Network plot for OS of nine different treatment regimens for patients. The lines represent direct comparisons within the RCTs. The line thickness indicates the number of RCTs included in each comparison. BRAF_MEK: combination of BRAF and MEK inhibitors; CTLA-4_Adju: combination of CTLA-4 blockade and an adjuvant; CTLA-4_chemo: combination of CTLA-4 blockade and chemotherapy; MEK_chemo: combination of a MEK inhibitor and chemotherapy; Adju: adjuvant; Chemo: chemotherapy

**Fig. 3:**
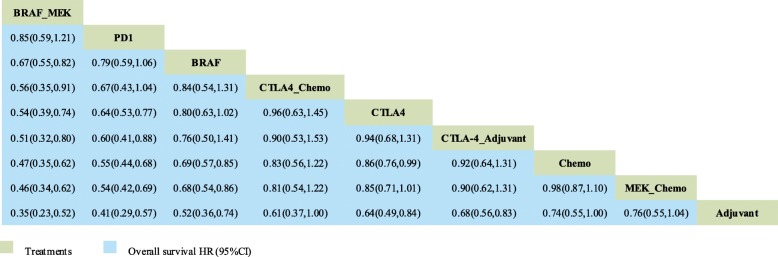
Network analysis of overall survival. The results of OS are expressed as HRs (95% CrIs). The data should be read from left to right

Twelve trials with 9 comparisons were analysed for OS (Fig. 2): PD-1/L1 blockade versus chemotherapy (1 trial, *N* = 418), CTLA-4 blockade versus chemotherapy (1 trial, *N* = 655), PD-1/L1 blockade versus CTLA-4 blockade (1 trial, *N* = 843), combination of CTLA-4 blockade and chemotherapy versus chemotherapy (1 trial, *N* = 502), combination of BRAF and MEK inhibitors versus BRAF inhibitor (3 trials, *N* = 1622), combination of MEK inhibitors and chemotherapy versus chemotherapy (5 trials, *N* = 1368). Figure 3 shows that the combination of BRAF and MEK inhibitors had a survival advantage compared with all the other treatments except PD-1/L1 blockade (HR: 0.85; 95% CrI: 0.59, 1.21). PD1/L1 blockade had a better efficacy than the CTLA-4 blockade (HR: 0.64; 95% CrI: 0.53, 0.77) and CTLA-4 blockade combination with an adjuvant (HR: 0.60; 95%, CrI: 0.41, 0.88).

### Progression-free survival

**Fig. 4:**
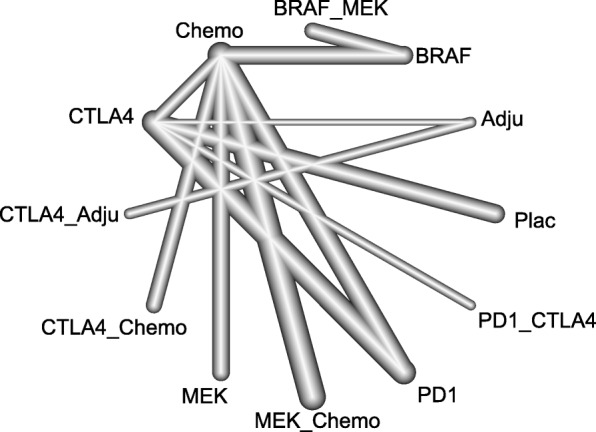
Network plot for the PFS of eleven different treatment regimens for patients. The lines represent direct comparisons within the RCTs. The line thickness indicates the number of RCTs included in each comparison. BRAF_MEK: combination of BRAF and MEK inhibitors; CTLA-4_Adju: combination of CTLA-4 blockade and an adjuvant; CTLA-4_chemo: combination of CTLA-4 blockade and chemotherapy; MEK_chemo: combination of a MEK inhibitor and chemotherapy; Adju: adjuvant; Chemo: chemotherapy; PD-1_CTLA-4: combination of PD-1/L1 and CTLA-4 blockade; Plac: placebo

**Fig. 5:**
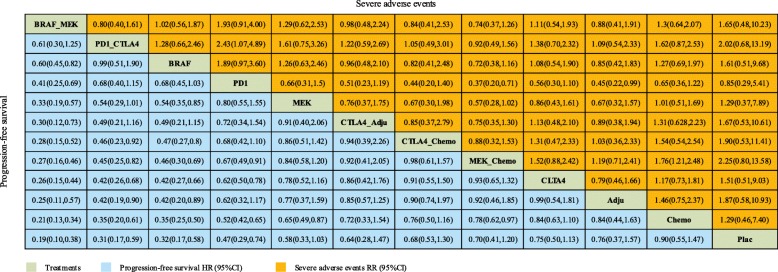
Network analysis of progression-free survival and severe adverse events. The results of PFS are expressed as HRs (95% CrIs), and severe adverse events are expressed as RRs (95% CrIs). The data should be read from left to right

PFS was analysed using the following eleven comparisons (Fig. 4): PD-1/L1 blockade versus chemotherapy (3 trials, *N* = 1363); CTLA-4 blockade versus chemotherapy (1 trial, *N* = 655); CTLA-4 blockade versus placebo (1 trial, *N* = 951); PD-1/L1 blockade versus CTLA-4 blockade (1 trials, *N* = 843); combination of CTLA-4 blockade and chemotherapy versus chemotherapy (1 trials, *N* = 502); combination of CTLA-4 blockade and placebo versus CTLA-4 blockade or placebo (1 trials, *N* = 502); combination of CTLA-4 blockade and PD-1/L1 blockade versus CTLA-4 blockade and PD-1/L1 blockade; combination of BRAF and MEK inhibitors versus BRAF inhibitor (3 trials, *N* = 1622); combination of MEK inhibitor and chemotherapy versus chemotherapy (5 trials, *N* = 1368); BRAF inhibitor versus chemotherapy (2 trials, *N* = 925); MEK inhibitor versus chemotherapy (3trials, *N* = 723). The results showed that the combination of BRAF and MEK inhibitors showed a significant advantage in PFS compared with all the other implements except the combination of PD-1/L1 blockade and CTLA-4 blockade (HR:0.61; 95% CrI: 0.30, 1.25) (Fig. 5). The combination of PD-1/L1 blockade and CTLA-4 blockade was superior to that of CTLA-4 blockade and chemotherapy (HR: 0.46; 95% CrI: 0.23, 0.92), and BRAF inhibitors showed a better survival benefit than MEK inhibitors (HR:0.54; 95% CrI: 0.35, 0.85).

### Safety and toxicity

Adverse events of grade 3 or higher (WHO≥G3) were reported in this study, and all of the details are presented in Fig. 5. According to the results, the combination of PD1/L1 blockade and CTLA-4 blockade showed a higher incidence of severe adverse events than the PD1/L1 blockade alone (RR: 2.43; 95% CrI: 1.07, 4.89). Additionally, the combination of MEK inhibition and chemotherapy was associated with a higher incidence of severe adverse events than chemotherapy (RR: 1.76; 95% CrI: 1.21, 2.48).

### Ranking analysis

Ranking analysis was performed using the P-score of OS and PFS. Concerning PFS, the combination of BRAF and MEK inhibitors was the best option for treatment (P-score = 0.99), followed by the combination of PD-1 blockade and CTLA-4 blockade (P-score = 0.86) and the BRAF inhibitor alone (P-score = 0.86).

Additionally, concerning OS, the combination of BRAF and MEK inhibitors was also the best option of treatment (P-score = 0.97), and adjuvant treatment seemed to be the least effective (P-score = 0.01).

### Assessment of heterogeneity and inconsistency

Network meta-analysis for PFS (tau-square = 0.037; I^2^ = 62.9%) showed high heterogeneity, and OS (tau-square = 0, I^2^ = 0) showed low heterogeneity. The tau-square estimates of PFS and OS are reported in Additional file 2. Inconsistency occurred in the network for PFS (*P* = 0.0003), but it was not statistically significant in the OS network meta-analysis (*P* = 0.98).

### Risk of bias

All the articles were assessed for the risk of bias by the Cochrane Risk of bias tool, and more than 50% of the trials were low risk in all seven biases, as shown in the Additional files 3 and 4. The data were extracted by Q.A. and Z.L. with predefined data collection forms. The extracted data were verified independently.

## Discussion

With the emergence of targeted therapies, the treatments of advanced melanoma have significantly improved, and recently, MAPK pathway inhibitors and immune checkpoint inhibitors have been shown to be effective therapeutic choices. However, comparative trials between these two categories are lacking, especially regarding the different combinations based on these two categories. We used network meta-analysis to explain the efficacy and toxicity of all available combinations and provide suggestions for patients and clinicians. In this study, we found that the combination of BRAF and MEK inhibitors showed a significant advantage in PFS compared with all of the other implements except for the combination of anti-PD-1/L1 inhibitors and anti-CTLA-4 inhibitors (HR:0.61; 95% CrI: 0.30, 1.25). Regarding OS, this combination was also superior to the other modalities except for PD-1/L1 inhibitors (HR: 0.85; 95% CrI: 0.59, 1.21). Because the difference between the combination of BRAF and MEK inhibitors and combination of PD-1/L1 and CTLA-4 inhibitors showed no significance, we deduced that these two treatments showed non-inferiority in PFS. Similarly, regarding OS, we inferred that the combination of BRAF and MEK inhibitors showed a non-inferiority relationship with PD-1/L1 inhibitors. Because the efficiency of treatment was also associated with the risk of toxic effects, we analysed the severe adverse event rates among different implements; the results showed that there was no modality with a lower rate than chemotherapy. The combination of a MEK inhibitor and chemotherapy was the only implement with a higher rate than chemotherapy (RR: 1.76; 95% CrI: 1.21, 2.48).

From our results, we determined that treatment with the combination of BRAF and MEK inhibitors was superior to each modality except the combination of PD-1/L1 blockade and CTLA-4 blockade in PFS, with no increased risk of toxicity than any of the other treatments. Additionally, the combination of BRAF and MEK inhibitors showed a greater benefit than any implement except for the PD-1/L1 blockade in OS. However, compared with the combination of PD-1/L1 and CTLA-4 inhibitors in PFS, the combination of BRAF and MEK inhibitors showed no significant difference, and indicated that these two categories are non-inferior. Again, in OS, the combination of BRAF and MEK inhibitors was also not better than PD-1/L1 blockade, with no increased severe adverse event rate. Our findings were supported by two similar network analyses that also demonstrated the efficacy of the combination of BRAF and MEK inhibitors [40, 41]. However, in contrast to the OS data, our results showed that the combination of BRAF and MEK inhibitors showed no significant difference with CTLA-4 and GM-CSF. With the PD-1/L1 blockade, this condition may have been due to the limited number of patients who had a *BRAF* mutation. Thus, only one trial of CTLA-4 and GM-CSF was included in their study and caused heterogeneity. In our study, we included more than one trial and grouped them according to their properties [41]. Additionally, within the toxicity data, our results showed no evidence of a higher risk of toxicity with the PD-1/L1 and CTLA-4 combination, not as reported in the previous study. In our analysis, the combination of a MEK inhibitor and chemotherapy was the only treatment with an increased risk of toxicity. This difference might be because we used only severe adverse events as an estimate of toxicity.

There are several advantages in our study. We considered all available comparisons based on immune check point inhibitors and MAPK pathway inhibitors, such as MEK inhibitors, and their combination with chemotherapy, which were unavailable in prior studies [40, 41]. These results could provide an overall perspective of the efficacy and toxicity of different combination regimens in patients with advanced-stage melanoma. We did not limit the population of patients and included trials of patients with *BRAF*-mutant and PD-L1 expression [11, 35, 38, 42] because the *BRAFV600E* mutation was present in almost 40–60% of all patients with advanced melanoma, and *BRAF*-mutant patients could benefit from both MAPK pathway inhibitors and immune checkpoint inhibitors [43, 44]. We also included patients who received prior treatments because, in clinical practice, patients with treatment-naïve and prior treatments both exist. Thus, we believed the analysis of patients without limitations was more proper. Besides PFS, we also analyzed OS to evaluate the efficacy of different combination regimens for treatment of advanced-stage melanoma, which was not provided in the previous study [40]. We performed safety/toxicity analysis according to the rates of any SAEs because these results more realistic and practical than those of treatment-related adverse event rate and provided comprehensive insights into comparisons of the crossover for each treatment.

### Limitations

Our study also has several limitations. Clinical and methodological diversity/heterogeneity always exists across different clinical trials. Although we used unified inclusion criteria for eligible trials, these diversities could not be avoided. Especially because we included patients who had PD-L1 expression and a *BRAF* mutation, which might have introduced a bias when the results of the different treatments were compared in the network meta-analysis; patients with expression of different molecular biomarkers may have essentially different backgrounds. However, it is quite controversial whether the *BRAF* mutational status has a prognostic effect in advanced melanoma [45]. Similarly, regarding PD-L1 expression, there is insufficient evidence to prove its prognostic function, which consequently balanced the related risk of bias [44, 46, 47]. Heterogeneity was discovered in PFS analysis (tau-square = 0.037, I^2^ = 62.9%), but not found in OS analysis (tau-square = 0, I^2^ = 0). According to the results, heterogeneity existed in the comparison between MEK inhibitors and chemotherapy; we considered the heterogeneity in the different populations included in this comparison: one trial specifically limited the *BRAF*-*V600*-mutant population. However, like the results from primary studies, we had insufficient evidence to prove that such trials should be excluded even if they could lead to heterogeneity.

## Conclusions

Our network analysis offers the most comprehensive comparisons based on targeted and immune check point inhibitor therapy in patients with advanced melanoma without a mutant-status limitation, which is convincing than imposing the limitation. As in the absence of a direct comparison among different treatments, our results suggest that PFS is best in patients treated with the combination of BRAF and MEK inhibitors or the combination of PD-1/L1 and CTLA-4 blockade, the efficacy of these two treatments shows no significant difference. Meanwhile, OS is best with the BRAF and MEK inhibitors combination or PD-1/L1 inhibitor, with no significant difference between these two treatments. Additionally, because of heterogeneity and the limitations, this conclusion should be interpreted very cautiously. Furthermore, several direct comparisons are ongoing, such as BRAF-MEK inhibitors compared with PD-1/L1 or CTLA-4 blockade or PD-1/L1 in combination with a MEK inhibitor or BRAF inhibitor. We believe our results will be confirmed in future trials.

## Additional files

Additional file 1: **Table S1.** Characteristics of Trials. (DOCX 22 kb)

Additional file 2: **Table S2.** Q statistics for OS and PFS. (DOCX 13 kb)

Additional file 3: **Figure S1.** Risk of bias summary for each risk of bias item for each included study. (PDF 538 kb)

Additional file 4: **Figure S2.** Risk of bias graph for each risk of bias item presented as percentages across all included studies. (PDF 238 kb)

## Abbreviations

Adju: Adjuvant
ASCO: American Society of Clinical Oncology
Chemo: Chemotherapy
CrI: Credible interval
CTLA-4: Cytotoxic T-lymphocyte-associated antigen-4
ECCO: Annual Meetings and the European Cancer Conference
GM-CSF: Granulocyte-macrophage colony stimulating factor
HR: Hazard ratio
MAPK: Mitogen-activated protein kinase
NA: Not available
NMA: Network meta-analysis
OS: Overall survival
PD1/L1: Programmed-death-1/ligand-1
PFS: Progression-free survival
Plac: Placebo
PRISMA: Preferred Reporting Items for Systematic Reviews and Meta-Analyses
RCT: Randomized controlled trial
SAE: Serious adverse event
