# Supplementary material for: Comparative efficacy and safety of combination therapies for advanced melanoma: a network meta-analysis
Source: BMC Cancer. 2019 Jan 9;19:43. doi: 10.1186/s12885-018-5259-8 (PMC6327485; doi:10.1186/s12885-018-5259-8)
Supplement: Supplementary file 1 — Table S1. Characteristics of Trials. (DOCX 22 kb) [file 12885_2018_5259_MOESM1_ESM.docx]

**Table S1 Characteristics of Trials**

| Study | Phase | Regimes | | PD-L1 expression  N (%) | | *BRAF* V600  N (%) | | ECOG (%) | |
| --- | --- | --- | --- | --- | --- | --- | --- | --- | --- |
|  |  | Treatment group | Control group | Positive | Negative | Wild | Mutant | 0-1 | ≥2 |
| CheckMate 066 | 3 | 3mg/kg nivolumab+placebo | 1000 mg/m^2^  dacarbazine+placebo | 148  (35.4) | 270  (64.6) | 406  (97.1) | 0  (0) | 99 | 1 |
| CheckMate 037 | 3 | 3 mg/kg nivolumab | 1000 mg/m^2^dacarbazine  or carboplatin AUC-6  +175mg/ m^2^paclitaxel | 201  (50) | NA | NA | 89  (22) | 100 | 0 |
| KEYNOTE 002 | 2 | 2 mg/kg pembrolizumab  or 10 mg/kg pembrolizumab | Paclitaxel +carboplatin, paclitaxel alone, dacarbazine, or oral  temozolomide | NA | NA | 138  (77) | 41  (23) | 100 | 0 |
| NCT00257205 | 3 | 15mg/kg tremelimumab | 1000 mg/m^2^dacarbazine | NA | NA | NA | NA | 98 | NA |
| EORTC 18071 | 3 | 10 mg/kg ipilimumab | Placebo | NA | NA | NA | NA | 100 | 0 |
| KEYNOTE 006 | 3 | 10mg/kg pembrolizumab | 3 mg/kg ipilimumab | 671  (81) | 150  (18) | 525  (63) | 302  (36) | 100 | 0 |
| NCT00324155 | 3 | 10mg/kg ipilimumab  +850mg/m^2^  dacarbazine | 850 mg/m^2^dacarbazine  +placebo | NA | NA | NA | NA | 97 | 3 |
| NCT00094653 | 3 | 3mg/kg iplimumab  +1mg gp100 vaccine | 3 mg/kg ipilimumab  or 3 mg/kg ipilimumab | NA | NA | NA | NA | 98 | NA |
| NCT01134614 | 2 | 10mg/kg Ipilimumab + 250 μg Sargramostim | 10mg/kg Ipilimumab | NA | NA | NA | NA | 100 | 0 |
| CheckMate 069 | 2 | 3 mg/kg ipilimumab+  1 mg/kg nivolumab | 3 mg/kg ipilimumab+placebo | NA | NA | 108  (77) | 32  (23) | 98 | 1 |
| CheckMate 067 | 3 | 3 mg/kg ipilimumab+  1 mg/kg nivolumab | 3 mg/kg ipilimumab+  placebo or  3 mg/kg nivolumab  +placebo | 223  (24) | 620  (66) | 647  (69) | 298  (32) | 100 | 0 |
| coBRIM^*^ | 3 | 960mg vemurafenib+  60mg cobimetinib | 960mg vemurafenib+  placebo | NA | NA | 495  (100) | 0  (0) | 100 | <1 |
| NCT01584648^*^ | 3 | 150mg dabrafenib+  2mg trametinib | 150mg dabrafenib+  placebo | NA | NA | 423  (100) | 0  (0) | 100 | 0 |
| NCT01597908^*^ | 3 | 150mg dabrafenib+  2mg trametinib | 150mg dabrafenib+  placebo | NA | NA | 697  (100) | 0  (0) | 100 | 0 |
| NCT00110994^#^ | 2 | 400mg Sorafenib+  1,000mg/m^2^dacarbazine | 1,000mg/m^2^  dacarbazine | NA | NA | NA | NA | 100 | 0 |
| NCT00111007^#^ | 3 | 400mg Sorafenib+  225 mg/m^2^  Carboplatin+paclitaxel | 225 mg/m^2^  Carboplatin+paclitaxel | NA | NA | NA | NA | 100 | 0 |
| NCT00110019^#^ | 3 | 400mg Sorafenib+  225 mg/m^2^  Carboplatin+paclitaxel | 225 mg/m^2^  Carboplatin+paclitaxel | NA | NA | NA | NA | 100 | 0 |
| NCT00936221^#^ | 2 | 75mg selumetinib+  1,000mg/m^2^dacarbazine | 1,000mg/m^2^dacarbazine | NA | NA | NA | NA | 100 | 0 |
| EudraCT^#^2009-018153-23 | 2 | 75mg selumetinib+  75 mg/m^2^ docetaxel | 75 mg/m^2^ docetaxel | NA | NA | NA | NA | 100 | 0 |
| BRIM-3^#^ | 3 | 960mg vemurafenib+ | 1,000mg/m^2^dacarbazine | NA | NA | NA | NA | 100 | 0 |
| NCT01227889^#^ | 3 | 150mg dabrafenib+ | 1,000mg/m^2^dacarbazine | NA | NA | NA | NA | 67.2 | NA |
| NCT01245062^#^ | 3 | 2mg trametinib | 1,000mg/m^2^dacarbazine or  175mg paclitaxel | NA | NA | NA | NA | 100 | 0 |
| NCT00338130^#^ | 2 | 100mg selumetinib | 200mg/m^2^temozolomide | NA | NA | NA | NA | 98 | 2 |
| NCT01143402^#^ | 2 | 150mg/m^2^temozolomide | 1,000mg/m^2^Dacarbazine | NA | NA | NA | NA | NA | NA |

# : treatment with oral and intravenous ways; * :treatment with oral way; blank: treatment with intravenous way;

kg(kilogram);RECIST(Response Evaluation Criteria In Solid Tumors);NA: not available
