# Supplementary material for: Comparative efficacy and safety of combination therapies for advanced melanoma: a network meta-analysis
Source: BMC Cancer. 2019 Jan 9;19:43. doi: 10.1186/s12885-018-5259-8 (PMC6327485; doi:10.1186/s12885-018-5259-8)
Supplement: Supplementary file 2 — Table S2. Q statistics for OS and PFS. (DOCX 13 kb) [file 12885_2018_5259_MOESM2_ESM.docx]

Table S2. Q statistics for OS and PFS

| OS | | Q statistics | P value |
| --- | --- | --- | --- |
| Total | | 2.97 | 0.9821 |
| Within designs | | 1.70 | 0.9954 |
| Between designs | | 1.27 | 0.2602 |
| PFS | |  |  |
| Total | | 43.08 | 0.0003 |
| Within designs | | 42.67 | 0.0002 |
| Between designs | | 0.41 | 0.5211 |
| Within designs | |  |  |
| Treatment1 | Treatment2 |  |  |
| Adjuvant | CTLA4_Adjuvant | 0.04 | 0.8427 |
| BRAF | BRAF_MEK | 4.06 | 0.1314 |
| BRAF | Chemo | 0.73 | 0.3930 |
| Chemo | MEK | 24.66 | 0.0001 |
| Chemo | MEK_Chemo | 6.04 | 0.1964 |
| Chemo | PD1 | 3.82 | 0.2819 |
| CTLA4 | PD1 | 3.33 | 0.1892 |

chemo: chemotherapy
