# Supplementary material for: Comparative efficacy and safety of combination therapies for advanced melanoma: a network meta-analysis
Source: BMC Cancer. 2019 Jan 9;19:43. doi: 10.1186/s12885-018-5259-8 (PMC6327485; doi:10.1186/s12885-018-5259-8)
Supplement: Supplementary file 3 — Figure S1. Risk of bias summary for each risk of bias item for each included study. (PDF 538 kb) [file 12885_2018_5259_MOESM3_ESM.pdf]

	Random sequence generation (selection bias)	Allocation concealment (selection bias)	Blinding of participants and personnel (performance bias)	Blinding of outcome assessment (detection bias)	Incomplete outcome data (attrition bias)	Selective reporting (reporting bias)	Other bias
Carvajal 2014	+	+	+	+	?	?	?
Champion 2011	+	+	+	?	?	?	?
Eggermont 2015	+	+	?	+	?	+	?
Flaherty 2012	?	?	+	+	+	+	+
Flaherty 2013	+	+	+	+	?	?	+
Gupta 2014	+	+	+	+	?	?	?
Hauschild 2009	+	+	?	+	?	?	?
Hauschild 2012	+	+	+	+	+	+	+
Hodi FS 2010	+	+	+	?	+	+	+
Hodi FS 2014	?	+	+	+	+	?	+
Kirkwood 2012	+	+	+	+	?	+	+
Larkin 2014	?	?	+	+	+	+	+
Larkin 2015	?	?	+	+	+	+	+
Long 2014	+	+	+	+	+	+	+
McArthur 2014	+	+	+	+	+	+	+
McDermott 2008	+	?	?	?	?	+	+
Postow 2015	?	?	+	+	+	+	+
Ribas 2013	?	?	+	+	+	+	+
Ribas 2015	?	+	+	+	?	?	?
Robert 2013	+	+	+	?	?	?	?
Robert 2015	+	?	+	?	?	+	+
Robert C 2011	?	?	+	+	+	+	+
Robert C 2015	?	?	+	+	+	+	+
RobertL 2015	?	+	+	+	+	+	?
Weber 2015	+	+	+	+	?	+	?

+ low risk of bias; ? unclear risk of bias
