# Supplementary figures and images for: Comparative efficacy and safety of combination therapies for advanced melanoma: a network meta-analysis
Source: BMC Cancer. 2019 Jan 9;19:43. doi: 10.1186/s12885-018-5259-8 (PMC6327485; doi:10.1186/s12885-018-5259-8)

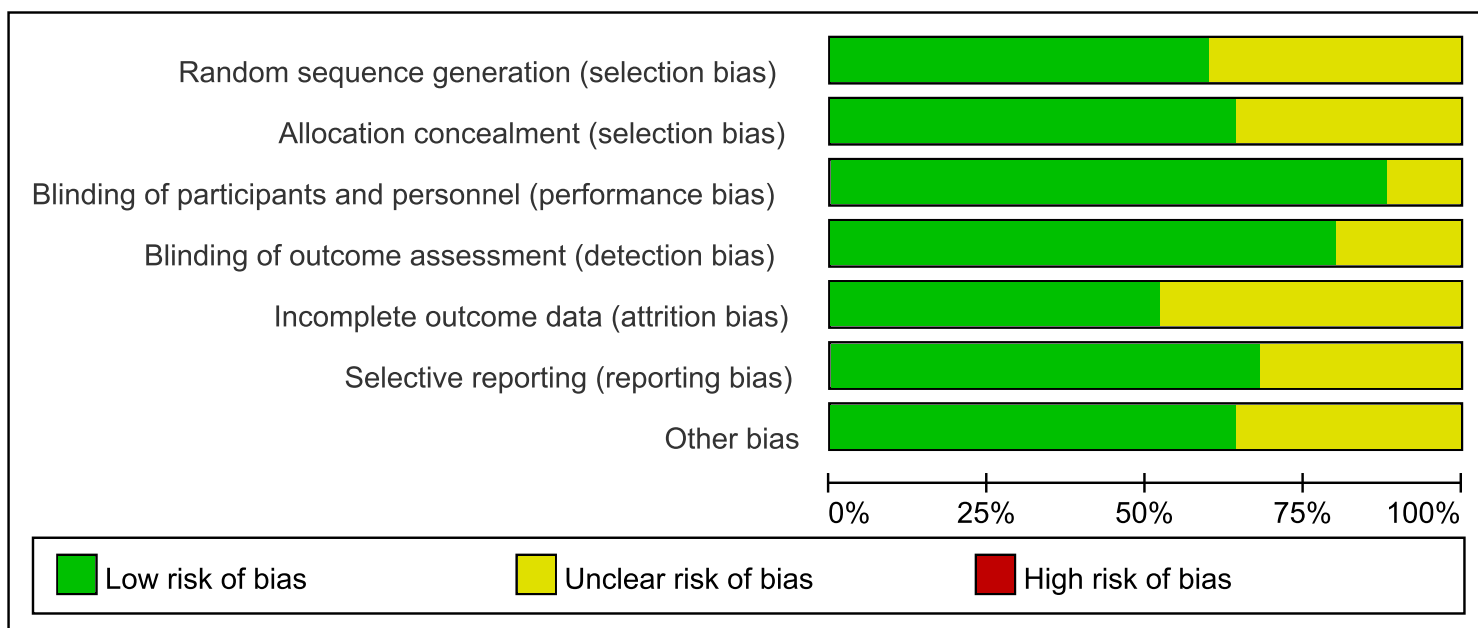

Supplement: Supplementary file 4 — Figure S2. Risk of bias graph for each risk of bias item presented as percentages across all included studies. (PDF 238 kb) [file 12885_2018_5259_MOESM4_ESM.pdf]
